# Neuroimaging through Sonolucent Cranioplasty: A Systematic Scoping Review Protocol

**DOI:** 10.3390/mps5050080

**Published:** 2022-10-09

**Authors:** Christina P. Rossitto, Alex Devarajan, Gabrielle Price, Muhammad Ali, Christopher P. Kellner

**Affiliations:** Department of Neurosurgery, Icahn School of Medicine, 1468 Madison Avenue, Annenberg Building, 8th Floor, New York, NY 10029, USA

**Keywords:** sonolucent cranioplasty, transcranial ultrasound, transcranioplasty

## Abstract

Cranioplasty is a neurosurgical procedure in which the skull bone is repaired after craniectomy. Recently, studies have suggested that sonolucent synthetic materials are safe and useful for cranioplasty. Sonolucent cranioplasty (SC) implants provide unprecedented opportunity in adult neurosurgery to monitor neuroanatomy, assess hemodynamics, view devices located within the implant, and conduct focused ultrasound treatments. Current research on SC includes proof-of-concept cadaveric studies, patient-related safety and feasibility studies, and case series demonstrating transcranioplasty ultrasonography (TCUS). The purpose of this protocol is to investigate the current literature on SC use and outcomes in TCUS. We will perform a systematic literature search following PRISMA-ScR guidelines. The search will be conducted using Ovid Embase, Ovid Medline, and Web of Science Core Collection databases. Titles, abstracts, and full texts will be screened. Joanna Briggs Institute critical appraisal tools will be utilized. Data extraction points will include subject characteristics, SC implant characteristics, ultrasound characteristics, and sonographic findings. These findings will provide a comprehensive review of the literature on sonolucent cranioplasty and directions for future research.

## 1. Introduction

Cranioplasty is a neurosurgical procedure in which the skull bone is repaired after craniectomy. Cranioplasty provides cosmetic benefits, protection from external atmospheric pressure distortion, and restoration of several critical physiologic processes. The ideal construct to correct cranial defects has evolved over time as several materials have been developed to replace the original skull bone [1].

In pediatric neurosurgery, open fontanelles provide a naturally occurring acoustic window for diagnostic ultrasound. However, fontanelle closure makes it impossible to leverage this method as a diagnostic tool in adult neurosurgery [2]. Sonolucent cranioplasty (SC) implants provide an unprecedented opportunity in adult neurosurgery to conduct neuromonitoring, evaluate hemodynamics, visually track devices located within the implant, and perform focused ultrasound treatments [2,3,4,5,6]. Current research on SC has utilized polymethylmethacrylate (PMMA), polyetheretherketone (PEEK), and polyolefin as sonolucent materials [7,8,9]. The first publication detailing diagnostic imaging through cranioplasty was published by Mursch et al. in 2018 using PEEK cranioplasty implants [9]. The term transcranioplasty ultrasound was first coined at Johns Hopkins University by Belzberg et al. in a proof-of-concept publication demonstrating TCUS through clear PMMA in a living patient and comparing clear PMMA, opaque PMMA, and PEEK in a cadaveric model [2]. This paper was quickly followed up by a second comparative study assessing transcranioplasty imaging through clear PMMA in a brain phantom [10]. Despite a large amount of clinical evidence supporting the use of cranioplasty and the frequency that these surgical procedures are performed, there is a dearth of knowledge about outcomes and risk factors associated with SC. Previous research on SC has been largely limited to proof-of-concept studies and case series [3,7,8].

Accordingly, this scoping review aims to analyze the trends, outcomes, risk factors, and characteristics of preceding investigations on transcranioplasty ultrasonography (TCUS) through SC in current neurosurgical practice.

## 2. Methods

### 2.1. Study Design

**Figure 1:**
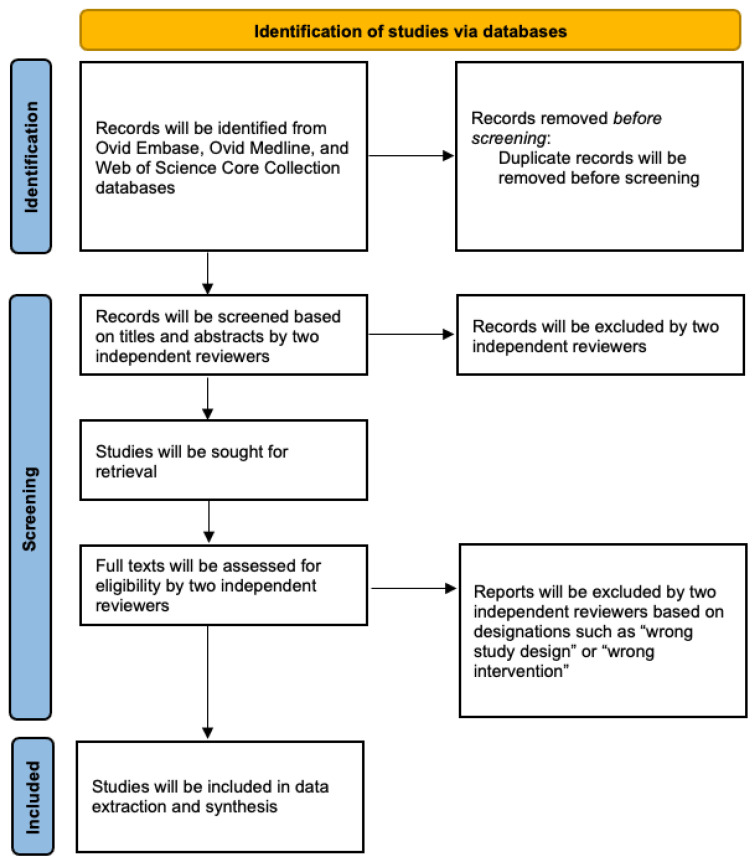
Flow diagram outlining the study search and selection.

The study selection and screening procedures are shown in Figure 1 [11]. The scope of this review will examine literature regarding the use of SC, among humans and bench top models, for the use of neuroimaging. Published full text articles, inclusive of case reports and series, which detail the new use of SC for diagnostic imaging will be considered. Manuscripts exploring the in vitro and in vivo characterization of sonolucent materials will be included. Studies detailing the use of SC for purposes other than neuroimaging and neuromonitoring will not be included for the review. Publications that are not original research articles (i.e., abstracts, review articles, and commentaries) will not be included. This systematic scoping review will adhere to PRISMA-ScR guidelines [12].

### 2.2. Participants

This scoping review will include manuscripts that involve neurosurgery patients who undergo TCUS through SC. There are no limitations on the types or number of participants included. Studies on bench top mdoels and animal models are also included.

### 2.3. Search Strategy

Ovid Embase, Ovid Medline, and Web of Science Core Collection databases will be searched to identify peer-reviewed articles characterizing the use of SC for preclinical in vitro and in vivo models and clinical patient studies (Table 1). Particularly, databases will be queried using search terms “clear”, “sonolucent”, “translucent”, “polymethyl methacrylate”, “polyolefin”, or “polyetheretherketone”, and “cranioplasty”. With this combination of broad search terms, we hope to encapsulate a vast array of manuscripts for our analysis.

### 2.4. Screening

Studies will be uploaded into Covidence for review and duplicates will be removed. Inclusion criteria will focus on (1) published full text articles, (2) which detailed new use of SC for the purpose of neuroimaging. Two reviewers will independently perform title and abstract screening of all studies. A full-text review will be performed by two reviewers. Any questions or disagreements during this process will be discussed and submitted to an additional supervising reviewer. The Covidence systematic review software will be used to evaluate inter-rater reliability.

### 2.5. Joanna Briggs Institute Critical Appraisal Tools

Article quality will be assessed using the Joanna Briggs Institute (JBI) critical appraisal tool for case reviews, case series, and quasi-experimental studies. An example of the JBI critical appraisal tool for case reviews is shown in ref [13]. JBI has published theories, protocols, and methodical processes for the critical appraisal and synthesis of several forms of peer-reviewed literature. The JBI critical appraisal tool was developed with the impetus to help improve clinical decision making in healthcare. Consequently, this tool will be used to critically appraise each article and evaluate its methodological quality. The purpose of this appraisal is to determine risk of bias, guarantee proper reporting and statistical analysis, and to assess the extent to which an investigation has addressed the potential for bias in its design, conduct, and analysis.

### 2.6. Data Extraction and Management

Two reviewers will independently extract data from included studies as follows: methods, characteristics of participants, interventions, and primary and secondary outcomes. Any disagreements on data extraction will be adjudicated through discussion with a third reviewer. The data items extracted by two independent investigators will include authors, date published, study design, prosthesis material, prosthesis brand, ultrasound transducer used, confirmation imaging, imaging artifact, imaging findings. For preclinical studies, the vessel used for experimental design will also be extracted. For clinical studies, sample size, sex, clinical indication for cranioplasty, prosthesis size and thickness, prosthesis location, dural substitute, and clinical complications will be extracted. 

## 3. Discussion

Cranioplasty is a critical neurosurgical procedure that provides cosmetic benefits, protection from external atmospheric pressure distortion, and restoration of several key physiologic processes such as glymphatic circulation, cerebral hemodynamics, and cellular mechanisms [14,15]. Harnessing the features of cranioplasty to create a sonolucent window offers several advantages over radiographic imaging, including reduced cost burden, no radiation exposure, ease of use, interactive image acquisition, and sequential monitoring. SC can be used for preventative and observational purposes which include but are not limited to sonographic detection of brain tumor recurrence, monitoring of cerebral blood flow and Doppler imaging of vessel collateralization, and measuring ventricular size for hydrocephalus [8]. Although our review focuses on the diagnostic imaging capabilities of TCUS, SC also can be used to perform several therapeutic techniques, including delineation of microbubbles with blood–brain barrier disruption to improve drug delivery and focused ultrasound ablation of brain malformations [7,16].

This review will discuss the most up to date literature on TCUS through sonolucent prosthesis. To our knowledge, this is the first systematic scoping review to assess the safety and effectiveness of TCUS using SC. This review will describe the landscape of SC experimental investigations and help to guide future studies. We will add to the literature by highlighting seminal studies that enhance our understanding of SC utilization and outcomes. 

This assessment may pose some limitations that must be taken into consideration. Because the literature applicable to this topic is sparse and largely includes case reports and case studies, these articles may lack detailed findings and consist of conclusions that are limited. The heterogeneous nature of the literature may also challenge data reporting. However, rigor will be maintained by utilizing PRISMA-SR guidelines, Covidence, and JBI critical appraisal. 

Altogether, this review will identify current evidence and gaps in knowledge on SC to motivate future research. Larger clinical trials are currently ongoing to evaluate TCUS in larger patient populations following extracranial–intracranial bypass, stroke, tumor, and external ventricular drain placement [3,17]. The hope is for this review to motivate controlled, quantitative data-focused clinical trials, which would enhance the likelihood that clinicians consider using sonolucent materials to perform cranioplasty. As these trials begin design, enrollment, and data analysis, this review will guide key variables in SC evaluation. By developing a framework for investigating SC, this investigation should serve as a gold standard reference describing the impact of SC in the field of neurosurgery. 

## Figures and Tables

**Table 1:** Electronic search plan for Ovid Embase, Ovid Medline, and Web of Science Core Collection.

**Embase Classic + Embase <1947 to date of search>** 1 ((clear or translucent or Sonolucent or PMMA or polymethyl methacrylate or polyolefin or polyether or polyetheretherketone or polymethylmethacrylate) adj3 (cranioplasty or cranioplasties or transcranioplasty or transcranioplasties or implant or prosthesis)).mp. 2 ((clear or translucent or Sonolucent) adj2 (polyether or polyolefin or PMMA or polymethyl methacrylate)).mp. 3 1 or 2
**Ovid MEDLINE(R) and Epub Ahead of Print, In-Process, In-Data-Review & Other Non-Indexed Citations and Daily <1946 to date of search>** 1 ((clear or translucent or Sonolucent or PMMA or polymethyl methacrylate or polyolefin or polyether or polyetheretherketone or polymethylmethacrylate) adj3 (cranioplasty or cranioplasties or transcranioplasty or transcranioplasties or implant or prosthesis)).mp. 2 ((clear or translucent or Sonolucent) adj2 (polyether or polyolefin or PMMA or polymethyl methacrylate)).mp. 3 1 or 2
**Web of Science Core Collection** TS = ((cranioplast*) NEAR/3 (clear or translucent or Sonolucent or PMMA or polymethyl or polyolefin or polyether or polyetheretherketone or polymethylmethacrylate)) OR TS = ((transcranioplast*) NEAR/3 (clear or translucent or Sonolucent or PMMA or polymethyl or polyolefin or polyether or polyetheretherketone or polymethylmethacrylate)) OR TS = (((implant) NEAR/3 (clear or translucent or Sonolucent or PMMA or polymethyl or polyolefin or polyether or polyetheretherketone or polymethylmethacrylate)) AND (brain OR crani* OR neuro*)) OR TS = ((prosthesis) NEAR/3 (clear or translucent or Sonolucent or PMMA or polymethyl or polyolefin or polyether or polyetheretherketone or polymethylmethacrylate)) OR TS = ((clear) NEAR/2 (polyether or polyolefin or PMMA or polymethyl)) OR TS = ((translucent) NEAR/2 (polyether or polyolefin or PMMA or polymethyl)) OR TS = ((Sonolucent) NEAR/2 (polyether or polyolefin or PMMA or polymethyl))

## Data Availability

Not applicable.
